# Lower limb kinetic comparisons between the chasse step and one step footwork during stroke play in table tennis

**DOI:** 10.7717/peerj.12481

**Published:** 2021-11-11

**Authors:** Yuqi He, Dong Sun, Xiaoyi Yang, Gusztáv Fekete, Julien S. Baker, Yaodong Gu

**Affiliations:** 1 Ningbo University, Ningbo, China; 2 Eötvös Lorand University, Budapest, Hungary; 3 Hong Kong Baptist University, Hong Kong, China

**Keywords:** Table Tennis, Kinetic, Lower limb, Footwork, Chasse step

## Abstract

**Background:**

Biomechanical footwork research during table tennis performance has been the subject of much interest players and exercise scientists. The purpose of this study was to investigate the lower limb kinetic characteristics of the chasse step and one step footwork during stroke play using traditional discrete analysis and one-dimensional statistical parameter mapping.

**Methods:**

Twelve national level 1 table tennis players (Height: 172 ± 3.80 cm, Weight: 69 ± 6.22 kg, Age: 22 ± 1.66 years, Experience: 11 ± 1.71 year) from Ningbo University volunteered to participate in the study. The kinetic data of the dominant leg during the chasse step and one step backward phase (BP) and forward phase (FP) was recorded by instrumented insole systems and a force platform. Paired sample T tests were used to analyze maximum plantar force, peak pressure of each plantar region, the force time integral and the pressure time integral. For SPM analysis, the plantar force time series curves were marked as a 100% process. A paired-samples T-test in MATLAB was used to analyze differences in plantar force.

**Results:**

One step produced a greater plantar force than the chasse step during 6.92–11.22% BP (*P* = 0.039). The chasse step produced a greater plantar force than one step during 53.47–99.01% BP (*P* < 0.001). During the FP, the chasse step showed a greater plantar force than the one step in 21.06–84.06% (*P* < 0.001). The one step produced a higher maximum plantar force in the BP (*P* = 0.032) and a lower maximum plantar force in the FP (*P* = 0) compared with the chasse step. The one step produced greater peak pressure in the medial rearfoot (*P* = 0) , lateral rearfoot (*P* = 0) and lateral forefoot (*P* = 0.042) regions than the chasse step during BP. In FP, the chasse step showed a greater peak pressure in the Toe (*P* = 0) than the one step. The one step had a lower force time integral (*P* = 0) and greater pressure time integral (*P* = 0) than the chasse step in BP, and the chasse step produced a greater force time integral (*P* = 0) and pressure time integral (*P* = 0.001) than the one step in the FP.

**Conclusion:**

The findings indicate that athletes can enhance plantarflexion function resulting in greater weight transfer, facilitating a greater momentum during the 21.06–84.06% of FP. This is in addition to reducing the load on the dominant leg during landing by utilizing a buffering strategy. Further to this, consideration is needed to enhance the cushioning capacity of the sole heel and the stiffness of the toe area.

## Introduction

As one of the most popular racket sports in the world, table tennis has always attracted much attention. With the increasingly strong competition of table tennis in international events, the technical requirements for table tennis players are getting higher and higher. Table tennis is a competitive sport carried out on a small playing area, which requires players to run continuously over a small range during a match. Players need to complete a series of instantaneous explosive actions and change directions quickly and frequently in the process of continuous movement to achieve the purpose of hitting the ball effectively (Francisco, Teresa & Vargas, 2005; Mansec, Dorel & Jubeau, 2018). Footwork is a necessary factor influencing the performance of table tennis players. Players perform large amounts of active running to ensure that they can reach the most suitable hitting position prior to playing the next stroke (Malagoli Lanzoni, Di Michele & Merni, 2007); this positive behavior can provide sufficient preparation time for playing the next stroke. There is a strong link between stroke, type of footwork, and different types of strokes that may be combined with specific types of footwork (Malagoli Lanzoni, Di Michele & Merni, 2014). Therefore, footwork is not only the basis but also one of the key points of table tennis training. The chasse step and one step are the basic footwork patterns that combine with forehand and backhand strokes in table tennis (Lam et al., 2018; Fuchs et al., 2018; McAfee, 2009; Zhang, Zhou & Yang, 2018). In addition, proficient mastery of footwork can bring advantages to energy transfer in the power chain of lower extremities. Therefore, the study of biomechanics in table tennis footwork is an interesting field for athletes and scientists.

The chasse step is a footwork movement that is used in combination with racket play to perform a set of defensive and offensive strokes by making easy side movements. The one step is a footwork movement which allows the player to move for relatively long distances in the shortest time possible (Malagoli Lanzoni, Di Michele & Merni, 2014). Several studies have investigated the biomechanical characteristics of footwork patterns during stroke play in table tennis. Lam et al. (2018) investigated the biomechanical information of ground reaction forces, plantar pressure, and joint kinetics distribution during topspin forehand under three typical footwork conditions. Shao et al. (2020) investigated the kinetics and kinematics differences between professional and novice athletes during one step footwork based on the Oxford foot model. In addition, the effect of foot performance during stroke-play has been demonstrated in previous studies. Qian et al. (2016) have identified the significant differences of in-shoe plantar pressure between different level table tennis players. One possible explanation for the differences observed is the synergy that exists between the torso and lower extremities during the entire stroke motion (Kasai & Mori, 1998). The energy generated by the lower limbs can be transferred to the upper limbs, significantly affecting the speed of the racket and ball (Seeley et al., 2011). The combination of footwork and hitting skills, through repeated practice, may add to the smooth transfer of energy through the kinetic chain.

However, kinematic, and kinetic analyses in recent table tennis studies only focus on the peak point data, this analysis method is widely accepted in research, but analyses only the peak data and could miss important temporal fluctuations that occur throughout the footwork phase. With the development of spatiotemporal variability in biomechanics, a more complete model framework is proposed (Xu et al., 2020; Hébert Losier et al., 2015; Smeets et al., 2019; Xu et al., 2021). Statistical parametric mapping (SPM) is a methodology that could test the statistical differences of continuous data such as kinematics and kinetics throughout the whole motion period to calculate accurately the significance threshold (Pataky, Robinson & Vanrenterghem, 2013; Pataky, 2010). Based on the one-dimensional characteristics of footwork movements changing with time, this study combined traditional discrete analysis with one-dimensional statistical parameter mapping (SPM 1d) to conduct statistical analysis on the lower limb kinetics data of table tennis players during the chasse step and one step footwork.

Therefore, the purpose of this study was to investigate the lower limb kinetic characteristics of the chasse step and one step footwork during stroke play using traditional discrete analysis and one-dimensional statistical parameter mapping (SPM 1d). The hypothesis of this study was that different footwork movements will demonstrate significant differences among traditional discrete variables, and that different footwork patterns will also show significant differences in the process of continuous changes with time. In addition, the results of this study will provide information for athletes and coaches to develop training programs and prevent foot injuries. The data will also provide reference information for the development and design of table tennis shoes and insoles.

## Materials & Methods

### Participants

Twelve national level 1 table tennis players from Ningbo University volunteered to participate in the study. All participants were free of any form of lower extremity injury or disease within 6 months prior to data collection. All participants were right-handed, had a dominant right leg, and were in good physical health. The Human Ethics Committee of Ningbo University approved the study (RAGH20200901). All participants received and signed written informed consent after being informed of the objectives, details, requirements, and procedures of the table tennis experiment. The demographic information of participants is displayed in Table 1.

**Table 1:** The demographic information table of the participant.

Population	Height (cm)	Weight (kg)	Age (year)	Experience (year)	Handedness
12	172 ± 3.80	69 ± 6.22	22 ± 1.66	11 ± 1.71	right

### Experimental design

**Figure 1:**
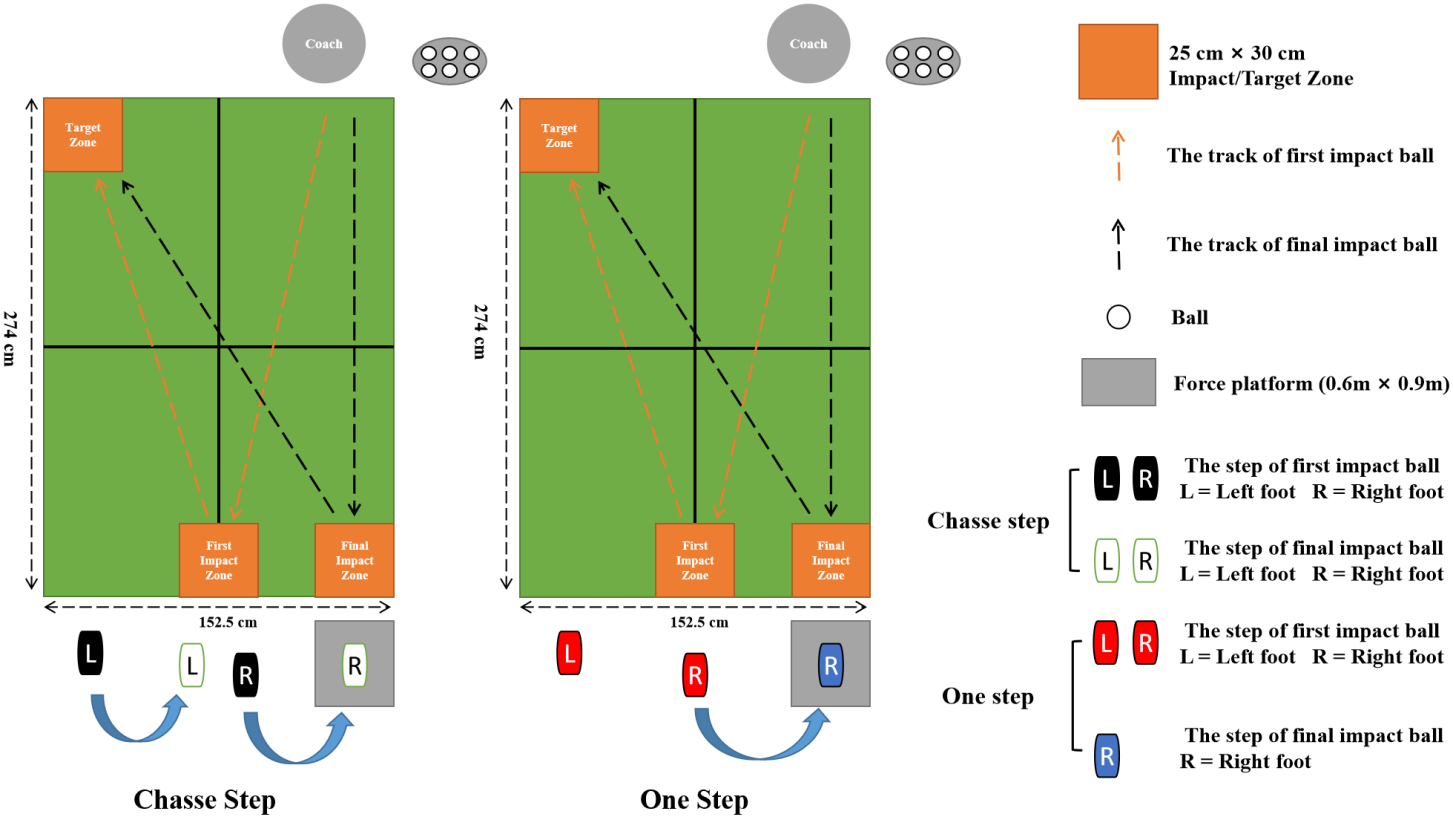
The experimental setting. The left side shows the chasse step, the right side shows the one step.

The experiment was performed at the Ningbo University table tennis training gymnasium. As outlined in Fig. 1, the kinetic data of the right leg was recorded using a Novel Pedar insole plantar pressure measurement system (Novel GmbH, Munich, Germany, sampling frequency of 100 Hz) and a force platform (AMTI, Watertown, USA, sampling frequency of 1,000 Hz). The table tennis table, balls, and rackets used complied with international standards. Prior to the start of the formal experiment, subjects were provided with time to warm up and familiarize themselves with experimental procedures. The warmup details included jogging on a treadmill at a comfortable speed and stretching. In the formal experimental, participants were asked to return the coach’s shot to the target area using chasse steps and one step, respectively. The hitting methodology for this experiment was as follows: the coach was asked to serve to the impact zone, which was in the centerline, and then serve to the impact zone which in the right side of the table tennis table. The player then needed to use the chasse step and one step footwork to return the ball to the target area. Participants were asked to complete four successful strokes using chasse step footwork in the first instance, then complete four further successful strokes using one step footwork. The smoothness of the movement was judged by the players themselves, and the quality and effect of the ball play were supervised by a qualified table tennis coach.

### Data collection and processing

**Figure 2:**
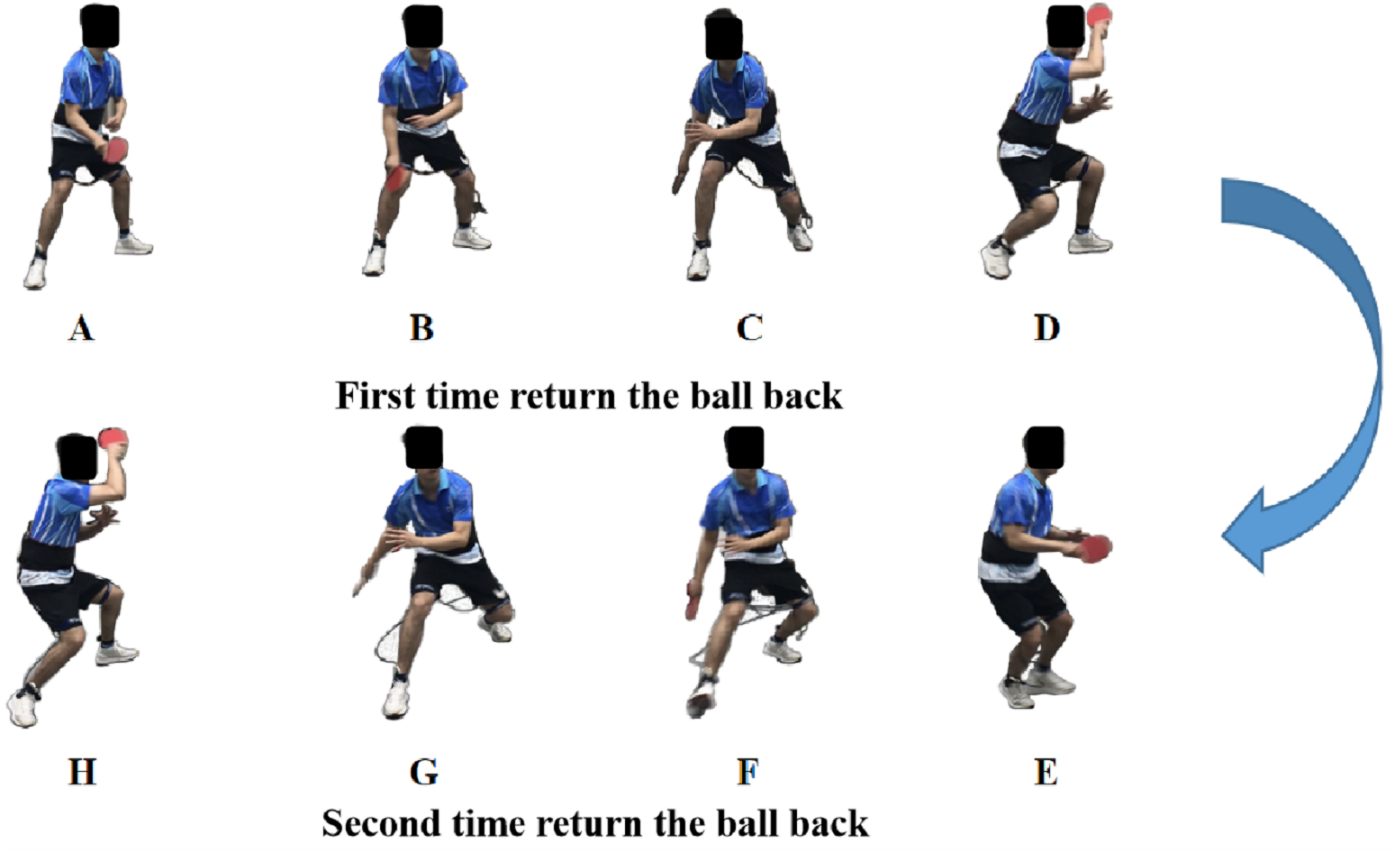
The technical performance of a participant during the test. (A–C) The backward phase (BP) of the first hit process. (C–D) The forward phase (FP) of the first hit process. (E–F) The backward phase (BP) of the second hit process. (G–H) The forward phase (FP) of the second hit process.

Information for plantar force, maximum plantar force, peak pressure of each plantar region, force time integral (FTI), and pressure time integral (PTI) were recorded by the Novel Pedar insole plantar pressure measurement system (Novel GmbH, Munich, Germany, sampling frequency of 100 Hz). The plantar was divided into six areas: Toe (T), Medial forefoot (MF), Lateral forefoot (LF), Midfoot (M), Medial rearfoot (MR), and Lateral rearfoot (LR). The data was then exported into MATLAB R2019a (The MathWorks, MA, United States), and a written script was produced to process the data. The participants remained in the ready position on the left side of the table, and the data collection started 1 s prior to the coach serving. The coach served after hearing the “start” command, the participant was asked to hit the ball with maximum force to the target zone. And data collection stopped after the participants completed the stroke action. As outlined in Fig. 2, in order to collect data closer to the real situation, in a data collection task, the coach will execute two serves, and the subjects are asked to complete two consecutive strokes. After completing the first stroke, the subjects were asked to complete the second stroke in combination with footwork. The footwork of the second stroke was asked to fully step on the force platform. And only the footwork of the second stroke was considered and analyzed. The contact period of the right leg of the selected footwork, from initial contact to take-off, is determined from the data provided by the force platform. As right leg movements were responsible for forgiving the greatest contribution to the forehand stroke (Lam et al., 2018). When the value of the ground reaction force reaches 10N for the first time, it is defined as the contact moment, and when the value of the ground reaction force decreases to 10N for the first time, it is defined as the airborne moment (Lam et al., 2018). By collecting the ground reaction of the right leg during forehand stroke motion through the force platform, two peaks can be observed. The first peak is the peak time of the right leg landing phase, and the second peak is the peak time of the right leg kicking phase. Based on the kinetics information of the force platform, the movement stages of the footwork movement were divided. The phase of ground reaction force from the 10N to the trough was defined as the BP (As shown in Figs. 2E–2F). And the phase of ground reaction force from the trough to below 10 N was defined as the FP (As shown in Fig. 2G–2H).

During SPM analysis processing, the generation of a separate integration curve was completed for each task before performing the SPM analysis. All kinetics data during the chasse step and one step footwork were extracted. The next step was to generate a custom MATLAB script and proceed with the interpolation process. The data points were expanded into a time series curve of 101 data points (representing 0–100% of the BP and FP phase) (Xu et al., 2020). For the traditional discrete variable analysis, a script was written, and analysis was performed using MATLAB to extract and calculate the data for maximum plantar force, peak pressure of each plantar region, FTI, and PTI of the chasse step and one step footwork during stroke play.

### Statistical analysis

Prior to statistical analysis, all data were tested using the Shapiro–Wilk normality test (*W* = 0.9361, *P* = 0.863). All traditional discrete variable analyses were carried out by SPSS 24.0 (SPSSs Inc, Chicago, IL, USA). Paired sample *T*-tests were used to analyze maximum plantar force, peak pressure of each plantar region, FTI, and PTI. In SPM analysis, the plantar force time series curve was marked as a 100% process. In addition, a paired-samples *T*-test in MATLAB was used to analyze plantar force between the chasse step and one step during BP and FP, respectively. An alpha level of 0.05 (α = 0.05) was set as being statistically significant.

## Results

**Figure 3:**
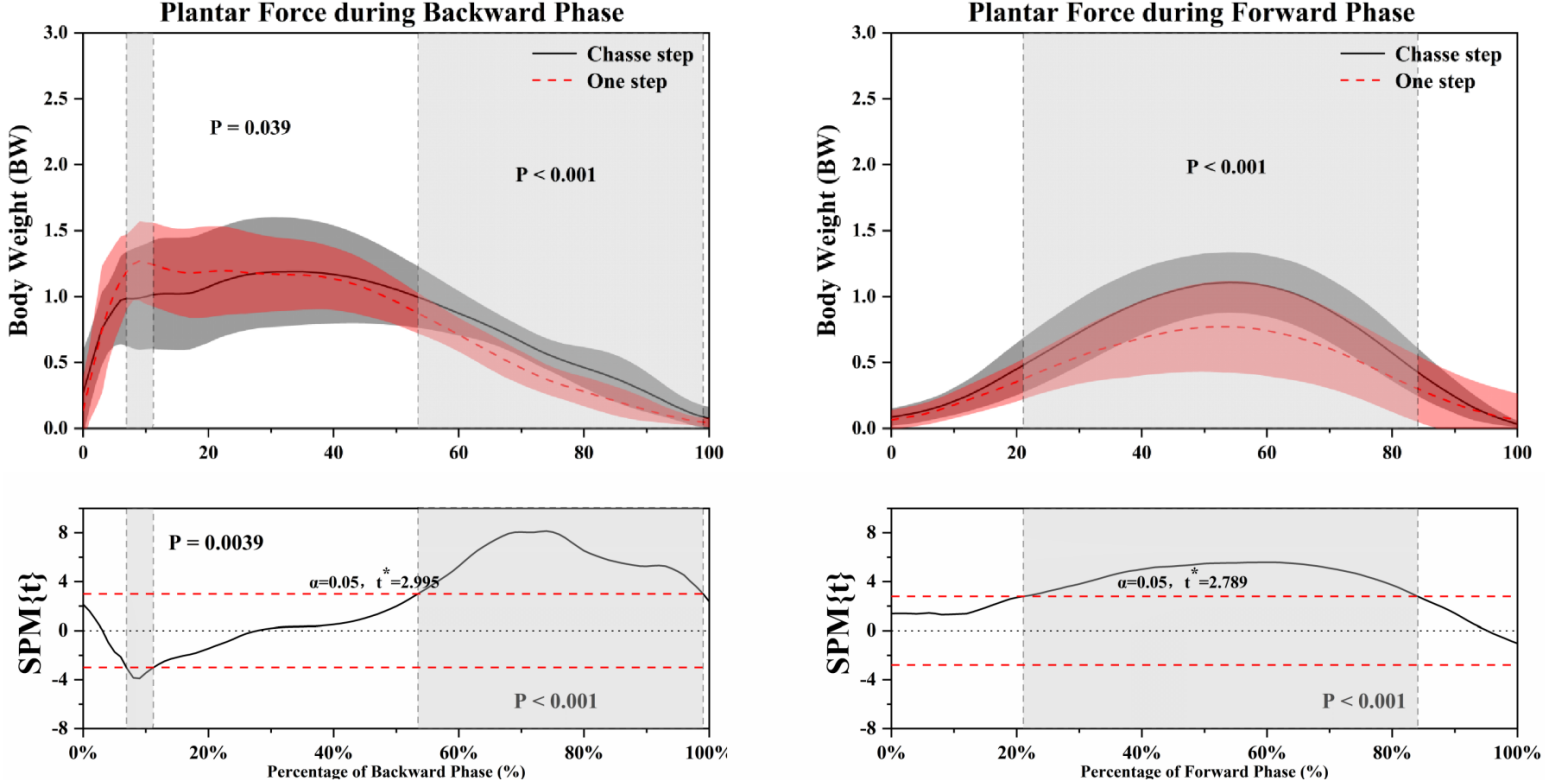
The statistical parametric mapping (SPM) results of plantar force between chasse step and one step during the backward and forward phase. Grey shaded areas indicate that there are significant differences (*p* < 0.05) between the chasse step and one step. Top figures refer to the comparison of plantar force between the chasse step and one step. The bottom figures refer to the details of Statistical Parametric Mapping (SPM) results. BW means body weight. “α = 0.05” means set the 0.05 as being statistically significant. “*” refers to significance with *p* < 0.05.

**Figure 4:**
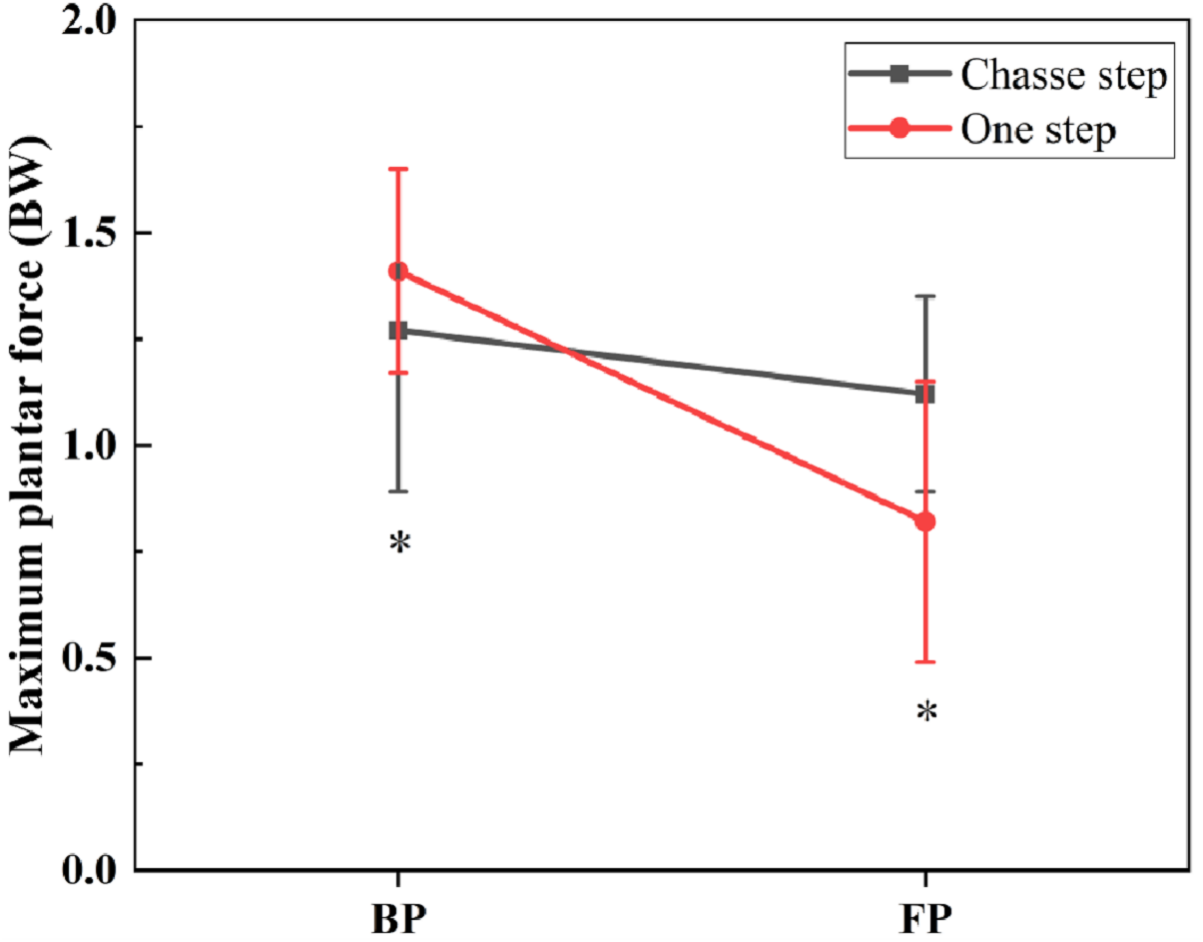
Comparison of maximum plantar force between chasse step and one step during BP and FP. BW means body weight. BP means backward phase, FP means forward phase. The asterisk (*) refers to significance with *p* < 0.05.

**Figure 5:**
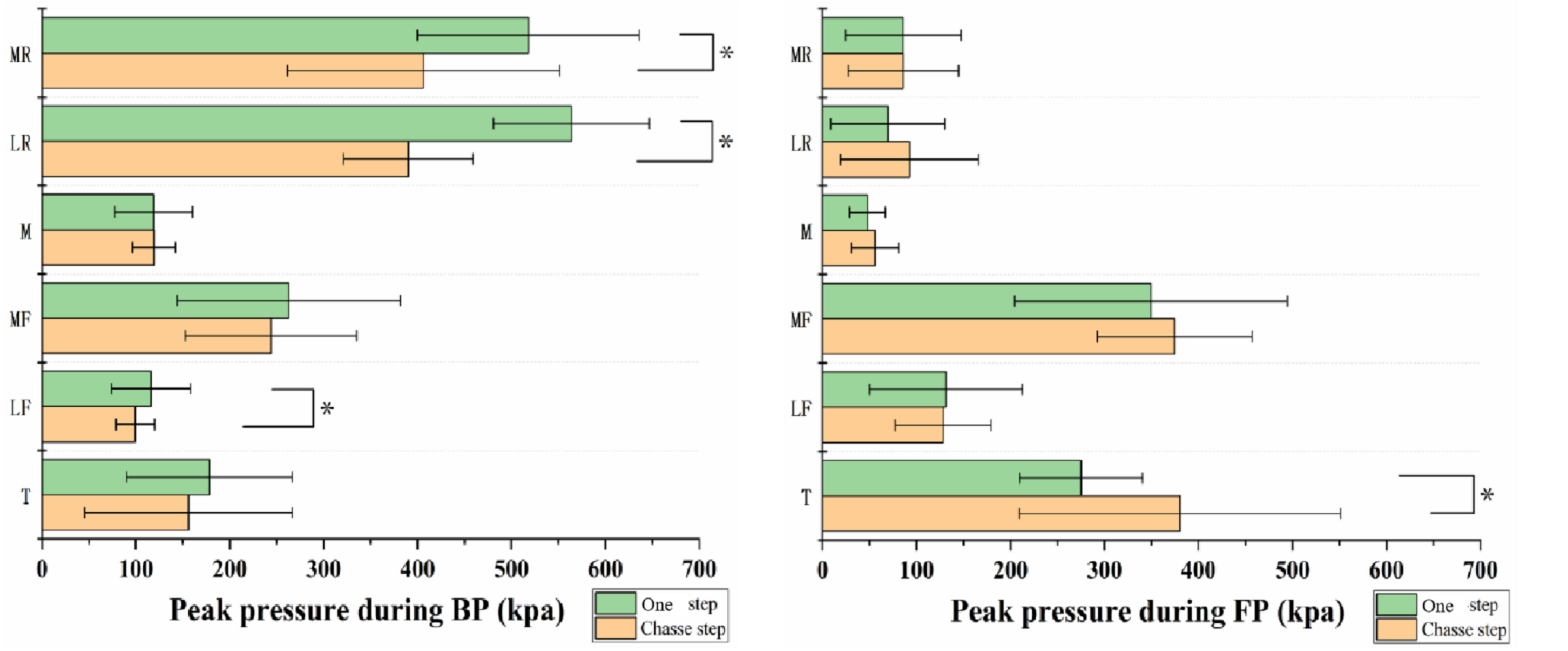
Comparison of peak pressure of each plantar region during BP and FP. The asterisk (*) refers to significance with *p* < 0.05. Toe (T), medial forefoot (MF), lateral forefoot (LF), midfoot (M), medial rearfoot (MR), lateral rearfoot (LR).

**Figure 6:**
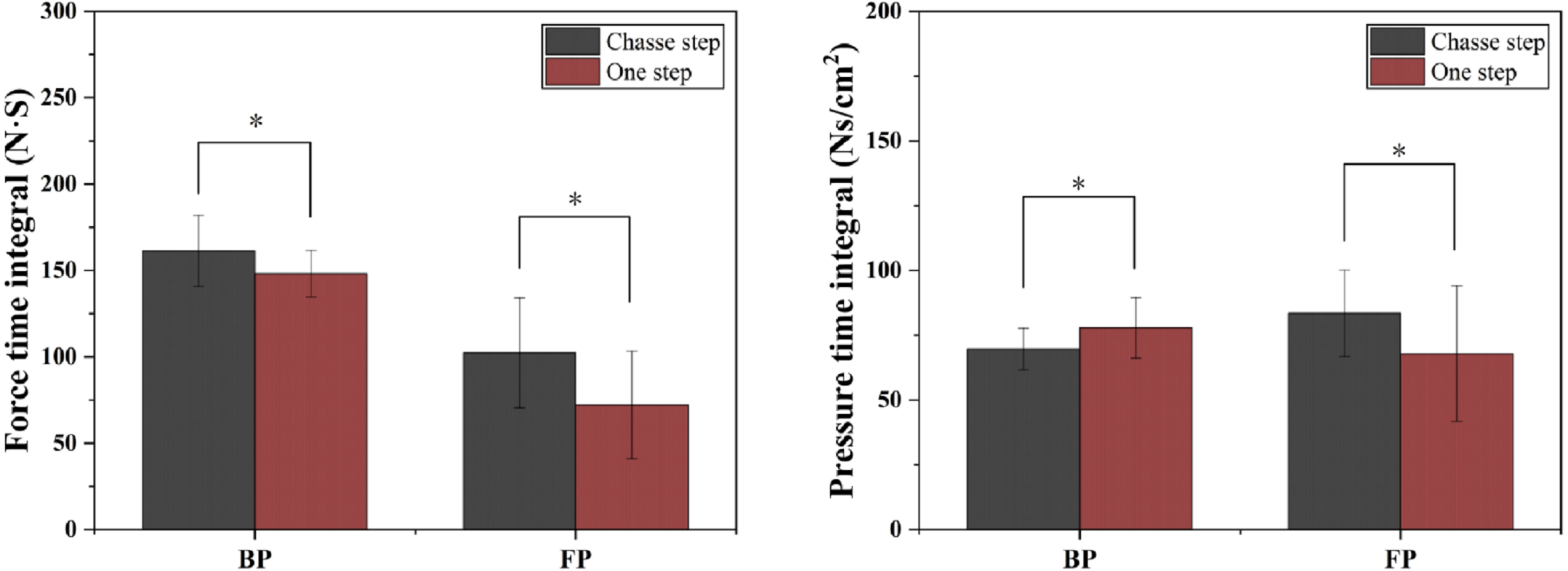
Comparison of PTI and FTI of plantar on driving foot between chasse step and one step during BP and FP. Left shows force time integral, right shows pressure time integral. “*” refers to significance with *p* < 0.05.

Figure 3 shows the difference in plantar force during BP and FP in a corresponding time series. Tables 2, 3, and 4 and Figs. 4, 5 and 6 outline the information related to traditional discrete statistical analysis.

### Plantar force

As shown in Fig. 3, the one step produced a greater plantar force than the chasse step during 6.92–11.22% BP (*P* = 0.039). The chasse step produced a greater plantar force than the one step during 53.47–99.01% BP (*P* < 0.001). During the FP, the chasse step showed a greater plantar force than one step in 21.06–84.06% (*P* < 0.001).

### Maximum plantar force

As shown in Table 2 and Fig. 4, the one step produced a greater maximum plantar force than the chasse step in the BP (*P* = 0.032). In addition, the chasse step produced a greater maximum plantar force in the FP (*P* = 0).

**Table 2:** The comparison of maximum plantar force during BP and FP between the chasse step and one step (unit: BW).

	Phase	Chasse step Mean ± SD	One step Mean ± SD	*P*-value
maximum plantar force	BP	1.27 ± 0.38	1. 41 ± 0.24	0.032^*^
	FP	1.12 ± 0.23	0.82 ± 0.33	0^*^

**Notes.**

BW body weight BP backward phase FP forward phase

* refers to significance with *p* < 0.05.

**Table 3:** The peak pressure comparison of each plantar region between the chasse step and one step at BP and FP (unit: kpa).

Partition	Phase	Chasse step Mean ± SD	One step Mean ± SD	*P* value
T	BP	174.97 ± 88.64	178.13 ± 89.03	0.742
	FP	388.85 ± 165.38	277.14 ± 59.61	0^*^
LF	BP	100.52 ± 20.74	116.04 ± 42.58	0.042^*^
	FP	129.44 ± 45.84	132.60 ± 83.07	0.764
MF	BP	243.75 ± 91.12	262.45 ± 114.63	0.069
	FP	379.43 ± 83.39	348.65 ± 145.31	0.078
M	BP	119.01 ± 23.56	119.01 ± 41.84	1.000
	FP	55.82 ± 24.29	47.71 ± 19.72	0.104
LR	BP	395.11 ± 64.81	563.72 ± 83.89	0^*^
	FP	90.43 ± 74.95	70.74 ± 61.52	0.206
MR	BP	404.27 ± 146.27	517.96 ± 119.44	0^*^
	FP	85.58 ± 57.32	85.17 ± 60.46	0.976

**Notes.**

BP backward phase FP forward phase T Toe MF Medial forefoot LF Lateral forefoot M Midfoot MR Medial rearfoot LR Lateral rearfoot

* Refers to significance with *p* < 0.05.

**Table 4:** FTI and PTI comparison between the chasse step and one step during BP and FP.

	Phase	Chasse step Mean ± SD	One step Mean ± SD	*P* value
FTI (N s)	BP	161.31 ± 20.73	148.13 ± 13.49	0^*^
	FP	102.29 ± 31.87	72.17 ± 31.04	0^*^
PTI (Ns/cm^2^)	BP	69.70 ± 7.98	77.91 ± 11.65	0^*^
	FP	83.49 ± 16.69	67.85 ± 26.14	0.001^*^

**Notes.**

FTI force time integral PTI pressure time integral BP backward phase FP forward phase

* Refers to significance with *p* < 0.05.

### Peak Pressure of each plantar region

As shown in Table 3 and Fig. 5, for the Toe, the chasse step produced a greater peak pressure than the one step in the FP (*P* = 0). In the LF, the one step produced a greater peak pressure than the chasse step during the BP (*P* = 0.042). In addition, the one step produced a greater peak pressure than the chasse step in the LR (*P* = 0) and MR (*P* = 0) during BP.

### FTI and PTI

As shown in Table 4 and Fig. 6, during BP, the chasse step produced a greater FTI (*P* = 0) and a lower PTI (*P* = 0) than the one step. During FP, the chasse step produced a greater FTI (*P* = 0) and PTI (*P* = 0.001) than the one step.

## Discussion

With the development of biomechanical measurement methods and techniques, biomechanical research of the lower limbs during table tennis has received extensive attention in recent years. The exploration of the lower limb kinetic mechanisms of footwork in table tennis can provide a theoretical basis for the optimization of the lower limb dynamic chain, the prevention of sports injury, and a contribution to the development of table tennis shoes. The purpose of this study was to investigate the differences in lower limb kinetic characteristics between the chasse step and one step footwork during stroke play in table tennis. The key findings of this study were that: (1) In 6.92–11.22% of the BP, the one step showed greater plantar force than the chasse step, and in 53.47–99.01% of the BP, the chasse step showed greater plantar force than the one step, which means that the one step showed greater plantar force on landing and that the chasse step showed a better force accumulation effect in the BP. In 21.06–84.06% of the FP, the chasse step showed greater plantar force than the one step, indicating better lower limb drive. (2) The one step was observed to have a higher maximum plantar force in the BP and a lower maximum plantar force in the FP compared with the chasse step. (3) The one step showed greater peak pressure in the MR, LR, and LF regions than the chasse step in the BP. In the FP, the chasse step showed a greater peak pressure in the T than the one step. (4) The one step showed lower FTI and greater PTI than the chasse step during the BP, and the chasse step showed greater FTI and PTI than the one step in the FP.

Compared with the one step, the chasse step showed greater plantar force in the 53.47–99.01% process of BP, and a greater plantar force during 21.06–84.06% process of FP, and a higher maximum plantar force in FP, as well as a greater peak pressure in the T. This, means that the chasse step shows greater complete lower limb extension and drive during the FP. It appears that the greater energy transfer promotes the generation of momentum (He et al., 2020; Ball & Best, 2007). As the origin of the dynamic chain, the lower limbs transfer the optimal activation energy from the lower limbs to the upper limbs through the continuous movement of the dynamic chain (Qian et al., 2016; Elliott, 2006). Lam et al. (2018) have investigated the biomechanical differences between different footwork during the topspin forehand in table tennis. In their study, the significantly higher peak pressures were in the plantar region of the total foot, toe, 1st, 2nd, and 5th metatarsal during chasse step and one step compared with one-step. The chasse step also showed a higher peak pressure than one step in the toe area. This is consistent with the results of this study. However, the MR, LR, and LF observed a higher peak pressure in the one step than the chasse step in this study, and this is not consistent with the results of Lam et al. (2018). This may be due to the different movement distances of the footwork resulting in different momentums resulting in different force values during landing. The chasse step showed higher peak pressure in the T than the one step. This could mean more plantarflexion during chasse step footwork in the FP. This may contribute to a greater range of weight transfer and thus momentum generation (He et al., 2020; Ball & Best, 2007; Zhang et al., 2017). Previous studies have reported on the underlying mechanisms of lower limb energy transfer and racket speed (Zhang et al., 2017; He et al., 2021; Iino & Kojima, 2009). In this study, the chasse step showed significantly greater plantar force than the one step in the 21.06–84.06% process of FP. From a practical point of view, athletes can enhance the plantarflexion function to bring greater weight transfer, resulting in a greater momentum during the 21.06–84.06% process of FP, thus improving the performance of racket speed. As well as from the perspective of sports monitoring, the quality of strokes during one step footwork can be monitored by analyzing the plantar force curves of players in the 21.06–84.06% process of FP.

PTI is a variable used to evaluate plantar load. This variable describes the cumulative effect of pressure over time in a certain area of the plantar. Excessive values may lead to tissue damage (Sauseng et al., 1999; Herman IJzerman et al., 2011). FTI is a variable that considers the integral of force overtime in a plantar area. PTI is the quotient of FTI divided by the contact area, which will provide an average cumulative load per square centimeter. PTI is better associated with plantar tissue injury than FTI (Herman IJzerman et al., 2011). In this study, the one step shows greater PTI than the chasse step during landing in the BP, and larger peak pressure was shown in MR, LR, and LF. This may have resulted in the center of gravity of the body being transferred to the dominant leg when landing, as well as being accompanied by the transfer of energy, leading to more load on the dominant leg during landing. Table tennis players rely more heavily on the movement of the dominant leg (Lam et al., 2018). Over-repetition coupled with high plantar pressure may result in injuries in athletes (Lam et al., 2018; Qian et al., 2016; Wong et al., 2007). Therefore, the athlete can reduce the load on the dominant leg during landing by practicing a buffer strategy. In addition, according to the results of this study, the design and material selection of table tennis shoes can be considered to enhance the cushioning capacity of the sole heel area and the stiffness of the toe area.

The key findings in this study not only provide information for exploring foot injuries of table tennis players but also provides reference information for the design and development of table tennis shoe soles. There are some limitations in the study that should be mentioned. Firstly, this study simulated the competition environment in the laboratory, which may have some differences from real competitions. Secondly, the experiment did not consider the foot morphology of the subjects, and different foot shapes may show different plantar load characteristics under the same footwork. In the future, biomechanical research related to the lower limbs of table tennis players should include the influence of foot morphology on experimental results. Real-time data and more advanced methods and equipment should be used to collect experimental information during a real competition environment.

## Conclusions

By combining traditional discrete analysis with the one-dimensional statistical parameter mapping (SPM 1d), we can reveal the kinetic differences of different footwork in a more comprehensive way. Significant differences in total plantar force between the chasse step and one step footwork were observed in 6.92–11.22% during BP, 53.47–99.01% during BP, and 21.06–84.06% during FP. Athletes can enhance the plantarflexion function to bring greater weight transfer, resulting in a greater momentum during the 21.06–84.06% of FP. The one step showed higher peak pressure in MR, LR, and LF, in BP, and the chasse step showed higher peak pressure at T and FP, indicating the potential design direction of shoes and insoles.

##  Supplemental Information

10.7717/peerj.12481/supp-1 Supplemental Information 1 Participant demographics

10.7717/peerj.12481/supp-2 Supplemental Information 2 SPM of plantar force

10.7717/peerj.12481/supp-3 Supplemental Information 3 Maximum Force

10.7717/peerj.12481/supp-4 Supplemental Information 4 Peak pressure

10.7717/peerj.12481/supp-5 Supplemental Information 5 Force time integral

10.7717/peerj.12481/supp-6 Supplemental Information 6 Pressure time integral

## Additional Information and Declarations

### Data Availability

The following information was supplied regarding data availability: The raw measurements are available in the Supplementary Files.
